# Distance Traveled and Cross-State Commuting to Opioid Treatment Programs in the United States

**DOI:** 10.1155/2011/948789

**Published:** 2011-07-06

**Authors:** Andrew Rosenblum, Charles M. Cleland, Chunki Fong, Deborah J. Kayman, Barbara Tempalski, Mark Parrino

**Affiliations:** ^1^National Development and Research Institutes, Inc. (NDRI), 71 W 23 Street, 8th Floor, New York, NY 10010, USA; ^2^College of Nursing, New York University (NYU), 726 Broadway, 10th floor, New York, NY 10003, USA; ^3^American Association for the Treatment of Opioid Dependence (AATOD), 225 Varick Street, 4th Floor, New York, NY 10014, USA

## Abstract

This study examined commuting patterns among 23,141 methadone patients enrolling in 84 opioid treatment programs (OTPs) in the United States. Patients completed an anonymous one-page survey. A linear mixed model analysis was used to predict distance traveled to the OTP. More than half (60%) the patients traveled <10 miles and 6% travelled between 50 and 200 miles to attend an OTP; 8% travelled across a state border to attend an OTP. In the multivariate model (*n* = 17,792), factors significantly (*P* < .05) associated with distance were, residing in the Southeast or Midwest, low urbanicity, area of the patient's ZIP code, younger age, non-Hispanic white race/ethnicity, prescription opioid abuse, and no heroin use. A significant number of OTP patients travel considerable distances to access treatment. To reduce obstacles to OTP access, policy makers and treatment providers should be alert to patients' commuting patterns and to factors associated with them.

## 1. Introduction

It has been well documented that geographical access is an important determinant of treatment utilization in the general population [1]. Among substance abusers longer travel distances are associated with shorter length of stay and lower probability of completion and aftercare utilization [2, 3]. Ongoing utilization (i.e., treatment retention) is especially important among methadone maintained patients because of the importance of continued medication that is often required to achieve and sustain treatment gains [4, 5]. Distance may be an especially relevant factor among methadone patients who live in rural or other areas where public transportation is limited. In addition to distance another geographical relevant factor is whether methadone patients cross state lines to attend an opioid treatment program (OTP), typically the only setting where methadone, for the treatment of opioid dependence, can be dispensed. 

The primary objective of the current study was to determine central tendency and variation in OTP enrollees' travel distance between home and the treatment program. A secondary objective was to identify program and patient factors associated with travel distance. Since, to our knowledge, this is the first study to describe commuting patterns among OTP enrollees, it is primarily descriptive. We did not have strong hypotheses regarding which patient or program characteristics are likely to be associated with travel distance. However, we expected that travel distance would be negatively correlated with urbanicity (since there is likely to be a higher concentration of OTPs within urban areas than nonurban areas). Also, based on findings from our previous study of OTP enrollees [6] we expected that factors associated with low urbanicity such as prescription opioid abuse, white race, employment, and first methadone treatment episode would also be associated with longer travel distances. The data collection protocol for this study was conceived and executed under the auspices of the Researched Abuse, Diversion and Addiction-Related Surveillance (RADARS) System. The RADARS System, which was initiated through a dialogue between the Food and Drug Administration (FDA) and Purdue Pharma L.P. and which is now an independent operation of Denver Health & Hospital Authority, is a proactive surveillance program that monitors and characterizes the abuse and diversion of opioid pain medication [7].

## 2. Methods

### 2.1. Sample

Data were collected from January 2005 through December 2009 from 23,141 enrollees in 84 OTPs located in 34 states. The number of study participants across the 84 OTPs ranged from 2 to 1,947 (median 155; quartile range: 71 to 372). Not all programs began study participation in January of 2005; 68 (81%) OTPs participated for 2 or more years; 12 (14%) participated for at least one year but less than two years; and 4 (5%) for less than one year. OTPs were selected primarily to represent regions in the USA where prescription opioid (PO) abuse was believed to be prevalent, for example, non-urban areas, especially those in the Appalachian region. Some OTPs were located in major metropolitan areas such as San Francisco and New York City, where PO abuse among OTP patients is believed to be relatively less prevalent. All programs were federally approved opioid agonist treatment programs and followed federal methadone treatment protocols that require an opioid-dependence diagnosis and an addiction history of at least 1 year [8]. The research protocol was approved by the Institutional Review Board of the National Development and Research Institutes, Inc. The respondents in this study include treatment seeking persons who reported abusing prescription opioids or heroin in the past 30 days and were not in methadone treatment in the previous 30 days. 

All patients enrolling in the study OTPs were targeted for recruitment. Several study procedures were implemented to maximize the number of OTP enrollees who participated. Patients were given an information sheet explaining the study's rationale and procedures and that participation was voluntary and anonymous. Patients who completed the one-page survey instrument were compensated with a $5 food coupon. The OTPs were instructed to have patients complete the survey during the first week of admission and to fax the surveys (along with a cover sheet that indicated the number of surveys included in the fax transmission) via a secure Internet-based fax service that forwarded the faxes to NDRI as an email attachment. (The faxed surveys were read by an automated form-driven data capture software program, Teleform Version 8.2, Verity, Inc., Sunnyvale, CA). In order to reinforce adherence to these procedures, each of the participating programs signed a letter of agreement that specified these study protocols and identified a liaison staff person who served as the principal contact between the OTP and the study's project director at the American Association for the Treatment of Opioid Dependence (AATOD; the organization responsible for communicating with the OTPs). The programs also received a stipend for their participation. Program participation was further reinforced by sending quarterly reports to each program of their data and an aggregate report of all study data. Further details of OTP participation and the subject recruitment protocol can be found in a 2007 publication that described differences between prescription opioid users and heroin users [6].

### 2.2. Variable Definitions

In order to assess travel distance and correlates associated with it, we utilized relevant data from the 1-page patient administered survey as well as program location information. Survey items addressed the following domains: demographics, treatment history, opioid use, drug craving, and pain; the survey also asked patients for their 5-digit ZIP code. Pain items were included because of the high prevalence of pain complaints among OTP patients [9, 10]. These measures are described below. 

#### 2.2.1. Travel Distance

The distance in miles between the center of the ZIP code of the patient's residence and the street location of the OTP was used to represent patient travel distance. MapPoint (Microsoft Corp., Redmond, WA) was used to determine the centroid point of the patient's residence ZIP code, the latitude/longitude of the OTP address, and the distance between these two locations. For statistical modeling, the natural logarithm of travel distance (with a constant of 1 added to all distances) was used. In order to avoid including subjects who may have written an incorrect ZIP code we excluded 266 patients who reported traveling more than two hundred miles to a treatment program. An examination of dispersion of the travel distance data showed that while distance was highly skewed to the right, the steepness of the decline precipitously dropped and remained at same level at approximately 200 miles. This pattern suggests that distances up to 200 miles were valid since they showed an expected pattern of fewer subjects with greater distance.

#### 2.2.2. United States Region

OTPs were classified as falling into one of four USA regions. These regions, as designated by the U.S. Census Bureau [11], are Northeast (Maine, New Hampshire, Vermont, Massachusetts, Rhode Island, Connecticut, New York, New Jersey, Pennsylvania); Midwest (Ohio, Indiana, Illinois, Michigan, Wisconsin, Minnesota, Iowa, Missouri, North Dakota, South Dakota, Nebraska, Kansas); South (Delaware, Maryland, District of Columbia, Virginia, West Virginia, North Carolina, South Carolina, Georgia, Florida, Kentucky, Tennessee, Alabama, Mississippi, Arkansas, Louisiana, Oklahoma, Texas); West (Montana, Idaho, Wyoming, Colorado, New Mexico, Arizona, Utah, Nevada, Washington, Oregon, California, Alaska, Hawaii).

#### 2.2.3. Beale Urbanicity Code

OTPs were in counties coded as high-density areas (population >1 million); moderately populated counties (≥250,000 and <1 million residents), and low populated counties (<250,000 residents); these three categories were determined by a modified version of the Beale Urbanicity Code [12]. The Beale code has 9 codes with 6 codes representing counties with <250,000 residents. Our previous research showed that a relatively small percent of respondents resided in the low populated counties; therefore we collapsed these 6 codes to reduce the skewness of the Beale distribution.

#### 2.2.4. Program and Patient Residence ZIP Code Areas

Both sizes of the ZIP code in which the patient resided and in which the program was located were indicated by area in square miles. For statistical modeling, the natural logarithm of square miles (with a constant of 1 added to all areas) was used.

#### 2.2.5. Bodily Pain a Reason for Enrolling

Patients were asked “Other than drug withdrawal pain, is bodily pain a reason for enrolling in methadone treatment?” with responses of “Yes” or “No.” 

#### 2.2.6. First Methadone Treatment

Patients were asked “Before coming to this program, when were you last in a methadone treatment program?” with a response of “Never” indicating first methadone treatment (Yes/No).

#### 2.2.7. Withdrawal Severity

Patients were asked “Which word best describes your drug withdrawal pain in the past week?” with five responses ranging from “None” to “Very Severe.”

#### 2.2.8. Urge to Use

Patients were asked “Which word best describes your urge to use your primary drug in the past week?” with five responses ranging from “None” to “Very Strong.”

#### 2.2.9. Recent Heroin Use

Patients were asked whether they had used heroin in the 30-day period before admission (Yes/No).

#### 2.2.10. Recent Prescription Opioid Use

Patients were asked whether they had used a prescription opioid to get high in the 30-day period before admission (Yes/No).

### 2.3. Data Analysis

A total of 5,349 respondents were not included in a multivariable mixed model analysis due to missing data on gender, race/ethnicity, source of income, urge to use, withdrawal severity, past methadone treatment, or pain as a reason for enrolling in treatment, resulting in a sample for multivariable analysis of 17,792 and a total sample of 23,141 OTP enrollees.

To account for clustering of the 17,792 patients in 84 OTPs, a linear mixed model analysis with a random intercept [13] was used to predict distance traveled to the treatment program by individual patients. The *nlme* package [14, 15] of the freely available, open-source *R* program [16] was used to fit linear mixed models. Program-level predictors included population density and the natural logarithm of the square miles of each OTP's ZIP code. Patient-level predictors included age, gender, race/ethnicity, employment, pain as a reason for enrolling in treatment, first methadone treatment, urge to use, withdrawal severity, use of prescription opioids in the past 30 days, and use of heroin in the past 30 days.

Predictors were entered in conceptually related blocks of variables. The first block included the program-level variables of urbanicity. The second block included the area of both the program ZIP code and the ZIP code of the patient's residence. The third block included the following demographic variables: age; gender; race/ethnicity; employment as the major source of income. The final block included the following characteristics related to each patient's opioid use: pain as a reason for enrolling in treatment; first methadone treatment; urge to use; withdrawal severity; prescription opioid use in the past 30 days; heroin use in the past 30 days. Each variable's relation to travel distance was estimated controlling for other variables on the same block and for all variables entered in previous blocks.

The use of a natural logarithm transformation effectively reduced positive skew in travel distance. To convey the effect of predictors in the mixed model analysis, fixed-effects regression coefficients were exponentiated (i.e., the Eulerian number, *e*, which is the base of the natural logarithm and approximately equal to 2.718282, was raised to the power of each regression coefficient). These exponentiated regression coefficients indicate how a one-unit change in the predictor multiplies travel distance. For example, a regression coefficient of .70 is interpreted as roughly a doubling (*e* ^.70^ = 2.014) of travel distance for a one-unit change in the predictor (or for a coefficient representing the contrast between a particular level of a categorical predictor and the reference category of that categorical variable).

In a separate analysis we used a multilevel logistic regression model to identify covariates among patients who crossed a state border to attend an OTP.

### 2.4. Mapping OTPs

We prepared a map of study and nonstudy OTPs to represent their location in the U.S. Location data for OTPs was obtained from the USA Substance Abuse and Mental Health Services Administration (SAMHSA) [17] including an updated report that there are no OTPs in South Dakota (Nick Reuter, SAMHSA, e-mail communication, Oct. 13, 2010). A map can immediately display spatial associations and patterns, otherwise impossible to identify in a tabular format. Data were imported into MapPoint (Microsoft Corp., Redmond, WA) to map the locations of OTPs and patient 3DZ code level data throughout the USA on a national map. Maps are traditionally a way of visualizing tabular data. 

## 3. Results

Table 1 presents data on the characteristics of patients and treatment programs. The area of the ZIP codes in which the 84 programs were located ranged from less than one square mile to more than one thousand squares miles, with a median of ten square miles. The number of patients responding to the survey in each program ranged from two to more than a thousand, with a median of 155 patients per program. More than half of all programs (54%) were located in densely populated urban areas, and at least fourteen programs were sampled from each USA region. 

Table 1 presents characteristics of patients and programs in the full sample (*n* = 23,141). Patient age ranged from 18 to 81 years with a median age of 32 years. Most patients were male and non-Hispanic white. Thirty percent of patients used both prescription opioids and heroin in the month before treatment. The comparably higher rate of prescription opioid use (73%) compared with heroin use (57%) may be accounted for by the oversampling of OTPs within regions were prescription opioid use is believed to be prevalent.

### 3.1. Travel Distance

**Figure 1:**
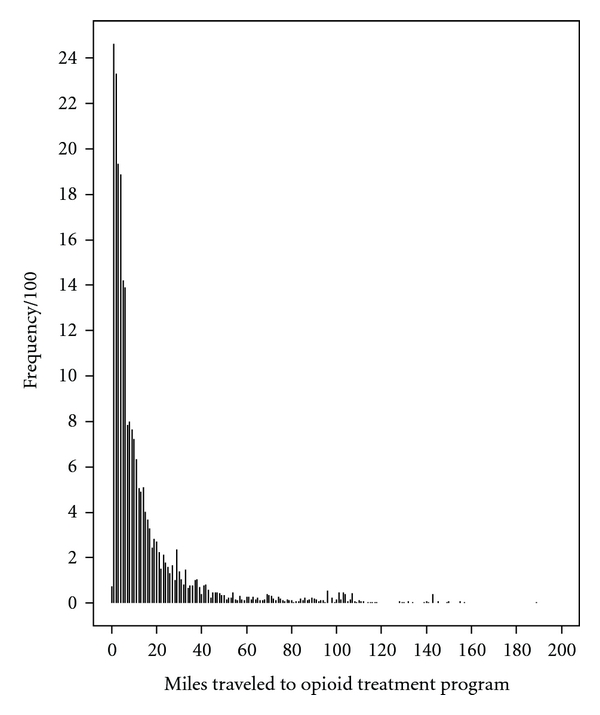
Distribution of travel distance (*n* = 23,141).

The average distance traveled from patients' residence to treatment programs was 15 miles, with a median travel distance of 7 miles and an interquartile range of 3 to 16 miles. More than half of all patients (60%) traveled less than 10 miles, and most patients (94%) traveled less than 50 miles; 26% of the patients traveled more than 15 miles and 2% traveled more than 100 miles to attend their OTP.  Figure 1 is a plot of travel distance (untransformed) which shows the central tendency, variability, and shape of the sample distribution in details (The mean and median travel distances were the same for the full sample (*n* = 23,141) and for the sample included in the linear mixed model analysis (*n* = 17,792)). 

### 3.2. Mixed-Effects Model for Travel Distance

Prior to controlling for program and patient level predictors we generated zero-order correlations of these variables with the log transformed value of distance. As can be seen in Table 2, most measures are significantly (*P* < .05) associated with distance. The strongest correlates (*r* ≥ .20) include urbanicity, ZIP code area of patient's residence, race/ethnicity, recent prescription opioid use, and no recent use heroin use. 

In the first block of the linear mixed model analysis, urbanicity was related to travel distance. Patients in moderately and low populated counties traveled 1.359 and 1.683 times as many miles as patients in densely population urban areas.

In the second block, the area of the ZIP code in which the patient resided was related to travel distance. A one-unit increase in the natural logarithm of square miles multiplied the travel distance by 1.360 miles.

In the third block, both age and race/ethnicity were related to travel distance. Patients between 44 and 81 years of age traveled 0.899 times as many miles as patients 18 to 29 years of age. Hispanics, African Americans, and patients in other racial/ethnic groups traveled 0.709, 0.635, and 0.896 times as many miles as non-Hispanic white patients.

In the fourth and final block, travel distance was positively related with prescription opioid use and negatively related with heroin use. Patients using prescription opioids in the month before enrollment traveled 1.085 times as many miles as patients not using prescription opioids.

We checked for multicollinearity in the model with all blocks entered, and all variance inflation factors were below 2.0, suggesting no problems.

### 3.3. Supplementary Analysis

Since frequency of racial/ethnic groups was strongly associated with urbanicity (e.g., percent non-Hispanic white in low, moderate, and high density areas was respectively, 93%, 89%, and 70%) a stratified analysis involved estimating the contrasts between patient racial/ethnic groups only for patients (*n* = 6, 902) enrolling in programs located in moderately or less populated counties (*n* = 39). Consistent with our hypothesis, African American patients traveled 0.679 and Hispanic patients traveled 0.852 times as many miles as non-Hispanic white patients (both *P* < .01).

### 3.4. Cross-State Commuting

Eight percent of all patients commuted to another state to attend an OTP, traveling an average of 50 miles (median = 36). More than 20% of the patients in 10 OTPs traveled across a state border to attend their program. Average (mean) distance traveled among these interstate patients from these 10 OTPs was 49 miles (median = 34). The average (mean) distance traveled among those same 10 OTPs who did not cross a state border was 25 miles (median = 14). An additional 4 OTPs had 5% to 19% of their patients commuting across state lines to attend the program. Cross-state commuting was more prevalent in the southeast (14% of patients) and Midwest (24% of patients) compared to the West (<1% of patients) and the Northeast (2% of patients). Among patients attending programs located in counties of different population densities, cross-state commuting was 8% in low, 15% in moderate, and 5% in high population density areas. 

#### 3.4.1. Correlates of Cross-State Commuting

Table 3 shows the results of the multilevel logistic regression analysis on cross-state commuting. Patients were more likely to cross a state border if their OTP was located in the Midwest (compared with the Northeast) and less likely if their OTP was in the West compared with the Northeast. Patients attending an OTP in relatively low densely populated areas (250 K − 1M ) compared with a metropolitan area (>1 M) as well as those whose OTP was located in a large ZIP code area were more likely to commute across a state border. The one significant patient covariate was race/ethnicity: African-Americans and other race/ethnicities (excluding Hispanics) were less likely to cross a state border than whites. The adjusted odds ratios for these same variables were also significant except for Midwest OTP location.

### 3.5. Location of Study OTPs

**Figure 2:**
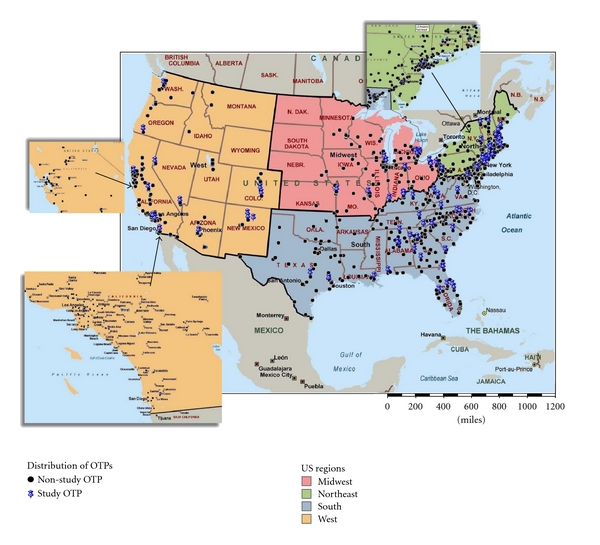
Distribution of opioid treatment programs (OTPs) within the continental United States: study OTPs = 83; nonstudy OTPs = 1130. Since the map only represents the continental USA it does not include the study OTP in Alaska or nonstudy OTPs outside of the continental USA.

Figure 2 displays a map representing the distribution of the participating OTPs (*n* = 84) to the distribution of nonparticipating OTPs (1,142) within the 4 US regions. Regionally, the respective distribution of study OTPs and all OTPs is Southeast (38% and 35%); Northeast (25% and 34%); Midwest (17% and 7%); West (20% and 24%). The respective Beale distribution of study OTPs and all OTPs is densely populated counties (>1M: 54% and 62%); moderately populated counties (250 K − 1M; 29% and 31%), and least densely populated counties (<250 K: 18% and 7%). Among the 12% (*n* = 2,878) of respondents who attended an OTP in a county with a different Beale code, 91% (*n* = 2,631) went to an OTP located in a more densely populated county. We also found that the more OTPs in a respondent's ZIP code the more likely they would travel within their ZIP code (OR = 14.9; CI = 13.3 to 16.8), within their state (OR = 7.5; 5.3 to 10.8) and a shorter distance (*β* = −.17, *P* < .001). 

### 3.6. Comment on Missing Data

Approximately three quarters (76%) of respondents had complete data. Missing data on a self-administered survey is not surprising. With the exception of Employment and First Methadone Treatment, each item was answered by >95% of the respondents. But since our multivariate approach was listwise deletion only those cases with complete data were retained in the multivariate analysis. Mean distance traveled for respondents with complete data is greater than distance traveled among respondents with missing data (15.4 versus 13.8 miles, *P* < .001). This does suggest some bias, although the magnitude of the distance between these two values is modest. Consistent with this finding, that a shorter travel distance is associated with missing data, we found that patient characteristics associated with missing data on other variables were also associated with most of the variables that were correlated with shorter travel distance (older age, nonwhite ethnicity, unemployment, previous methadone treatment, no prescription opioid use, and heroin use).

## 4. Discussion and Summary

### 4.1. Summary of Findings

Although the median distance traveled to an OTP was 7 miles, a small minority of patients (6%) traveled more than 50 miles to their OTP. Program variables related to patient travel distance included USA region and urbanicity. Patients enrolling in programs located in the Southeast and Midwest traveled greater distances than patients enrolling in the Northeast and West. Patients enrolling in programs located in moderately or low populated counties traveled greater distances than patients enrolling in densely populated urban areas.

Patient variables related to travel distance included age, race/ethnicity, and the type of opioid used in the month before enrollment. Younger patients traveled greater distances than older patients. Non-Hispanic white patients traveled greater distances than Hispanic, African American, and patients in other racial/ethnic groups. Patients using prescription opioids in the month before enrollment traveled greater distances than patients using heroin in the month before enrollment.

We also found that 8% of all patients crossed a state border to attend their OTP and that among a small number (10) of OTPs more than 20% of patients commuted across a state border. Minorities were less likely to cross a state border as well as patients whose OTP was located in the West (compared to the Northeast), in a metropolitan area or in smaller ZIP code area.

### 4.2. Discussion of Findings

Patients in the Southeast and Midwest traveled greater distances to treatment programs than patients in the Northeast. These regional differences cannot be entirely explained by regional differences in urbanicity, because urbanicity was included on the same block with USA region. Future research could determine whether individuals residing in the Southeast and Midwest regions travel greater distances because there may be proportionally fewer programs per opioid-dependent patient in those regions. We also found a lower likelihood of cross-state travel to an OTP located in the West versus Northeast (OR = .038). The small odds ratio suggests a large effect but the wide confidence interval (.002 to .839) suggests not much precision in estimating that effect. When these two regions are compared, commuting may be more common in the Northeast because smaller states are closer together and less common in the West because states are larger. The wide confidence interval (as well as the wide confidence interval for Midwest versus Northeast) is likely due to the small number of programs in the West and Midwest regions (see map) and the relatively small percentage of clients who commute across states to an OTP.

 Patients who enrolled in an OTP located in low or moderately populated counties traveled greater distances than patients in densely populated urban areas. (A similar pattern was also observed for cross-state commuting.) These differences may reflect the fact that travel distances for a variety of activities are greater when population density is lower. The youngest patients (18–29 years) traveled greater distances than the oldest patients (43–81 years). For older opioid-addicted individuals, other medical conditions may present more barriers to travel.

African American, Hispanic, and opioid users in other racial/ethnic groups traveled a shorter distance and (with the exception of Hispanics) were less likely to cross a state border when attending an OTP. Racial/ethnic minorities may have less access to transportation to a treatment program than non-Hispanic white patients. Also, it may be that more treatment programs are located in neighborhoods that are predominantly African American, Hispanic, or other non-white racial/ethnic groups. Travel distance can suggest more resources to travel a greater distance and possibly more motivation to overcome distance-related barriers. In that sense, travel distance may be both a sign of privilege and at the same time a burden and risk factor for treatment dropout. It should be noted that, though we know that non-Hispanic white OTP patients are more likely than patients from other racial/ethnic groups to reside in the Southeast and low population areas (two factors associated with greater commuting distance), these factors were controlled in the analysis and therefore do not entirely explain race/ethnic differences in distance traveled.

A possible reason why heroin use is associated with shorter travel distance is that heroin is generally more readily available in locations where OTPs are more densely located (i.e., urban areas). In nonmetropolitan areas heroin distribution markets are generally more informal and heroin is comparatively more expensive [18]. The association of prescription opioid abuse in less densely populated areas may partially be attributed to the relatively recent emergence of prescription opioids in rural areas [19] and the use of prescription opioids to self-medicate pain complaints, a phenomenon that has been observed in certain nonurban areas with a high rate of prescription opioid abuse, for example, Appalachia [20].

These data also have policy implications. In 2008 there were 268,071 OTP patients in the United States [21]. Given that 26% of patients traveled more than 15 miles to attend their OTP, that 6% of patients reside more than 50 miles from their OTP, and that among a subset of 10 OTPs more than 20% of patients traveled across a state border to attend their program, it is important for policy makers and treatment providers to be alert regarding commuting factors. Previous studies with other clinical populations report that distance is negatively associated with health care utilization. Although patients were only surveyed once (at enrollment) it is reasonable to assume that disparities in travel distance are also likely to be associated with treatment retention especially because federal law requires newly enrolled patients to attend the program at least 6 days a week to pick up their medication [22]; opioid-dependent patients who live at a greater distance from an OTP are likely to be at higher risk for dropping out of treatment than patients who live closer to a program. This may be especially evident in rural areas where OTPs are more dispersed, access to drug treatment is less available and underutilized [23], and where treatment continuity is likely to decline compared to treatment continuity in urban areas [24]. It is therefore important that programs respond to these challenges by assuring treatment continuity, such as establishing a more flexible take home policy, mobile methadone maintenance services, and methadone medical maintenance, that is, provision of methadone by an office-based physician or pharmacy [25, 26]. Efforts should also be made to provide low threshold treatment which may serve to attract and retain patients. Examples could include expansion of buprenorphine within OTPs along with establishing links with office-based physicians who prescribe buprenorphine. Office-based buprenorphine treatment is less stringently regulated than medication-assisted treatment provided at an OTP. Another concern is disaster planning. If local programs are wiped out by some natural disaster like hurricane Katrina, plans should be in place to bring mobile treatment to them, to set up programs in other facilities still standing that may not have offered treatment before, and to make sure that programs in neighboring states will promptly take in patients who are evacuated or who choose later on to relocate.

Although there are more than 1,200 OTPs throughout the United States it is important to recognize that OTP coverage varies across states [17]. For example, while some states such as New York, California, and North Carolina are relatively well saturated with OTPs (within certain regions), 3 states have no OTPs (North Dakota, South Dakota, and Wyoming), and there are a number of other states with only 1 to 3 operating OTPs, (e.g., Idaho, Mississippi, Montana, and Nebraska). Moreover various states impose moratoriums on the development of new OTPs from time to time and various states are (as of 2010) proposing or have recently passed extremely restrictive legislation with regard to the zoning of OTPs [27]. Despite evidence-based research demonstrating the effectiveness of methadone maintenance treatment [28, 29], expanding access to much-needed services for chronic opioid addiction through the OTPs has still not been realized. One means to address this limitation would be a sustained and national public education campaign about medication-assisted treatment.

### 4.3. Limitations

Travel distances were estimated based on the center of the ZIP code in which the patient resided and the exact location of the treatment program. If the exact locations of the patients' residence were known, travel distances could be estimated with greater precision. The straight line distance between the centroid of the patients' ZIP code to the exact location of the OTP does not capture other factors affecting travel time such as large variability in ZIP code size, road infrastructure, traffic, and mode of transportation. Also, travel cost and time may be offset if the treatment program location is close to resources which allow the patient to complete other life tasks such as going to work and shopping [30].

Because OTPs were selected to represent USA regions where prescription opioid use was expected to be prevalent, the sample is not representative of the population of OTP enrollees. Given the prevalence of prescription opioid use in less densely populated USA regions, the central tendency for travel distance observed in this sample may be higher than the central tendency for the population of OTP enrollees. The study did not formally determine the number of OTP enrollees. Therefore it is possible that not all patients who enrolled in an OTP during the study period completed a survey. However, ongoing conversations between the AATOD project director and the OTP liaison indicate that more than 90% of patients completed the survey. 

We recognize that there were several limitations to this study. However, we feel that it is important to present these results regarding travel distance to OTPs because it likely has a significant impact on the quality of life (including treatment retention) among patients. As far as we know this is the first study to document OTP travel distance and cross-state commuting across various locations throughout the United States. Further studies such as qualitative studies on how patients cope with long travel distances and longitudinal studies to examine the impact of distance on treatment retention should be conducted.

## Figures and Tables

**Table 1:** Opioid treatment program and patient characteristics.

	%	Mean	SD	Median	Minimum	Maximum
*Program Variables* (*n* = 84)						
USA Region						
Northeast	25					
Southeast	38					
Midwest	17					
West	20					
Beale urbanicity						
Metro Area >1 million	53					
≥250 K and <1 million	29					
<250 K	18					
ZIP code square miles		40	134	10	<1	1,186
Number of patients sampled		275	347	155	2	1,947

*Patient variables* (*n* = 23,141)						
Miles traveled to OTP		15	23	7	<1	200
ZIP code square miles		38	89	13	<1	5,031
Age						
18–29	43					
30–43	33					
44–81	24					
Male	60					
Race/ethnicity						
African american	9					
Hispanic	11					
White	77					
Other	3					
Employed	44					
First methadone treatment	50					
Pain a reason for treatment	34					
Strong urge to use	85					
Severe withdrawal	71					
Prescription opioid use past 30 days	73					
Heroin use past 30 days	57					

**Table 2:** Multilevel model predicting patient travel distance to OTP.

Predictor				95% Confidence interval		
	Zero-order correlation	Regression coefficient	SE	Lower	Est.	Upper
*Block one*						
USA Region	.21					
Southeast versus Northeast		0.592**	0.145	1.354	1.808	2.413
Midwest versus Northeast		0.354*	0.170	1.015	1.424	2.000
West versus Northeast		−0.215	0.169	0.576	0.807	1.130
Urbanicity	−.18					
250 K–1 M versus Metro >1 M		0.307**	0.129	1.051	1.359	1.758
<250 K versus Metro >1 M		0.521*	0.160	1.223	1.683	2.316

*Block two*						
Patient ZIP code area	.30**	0.307**	0.006	1.345	1.360	1.375
Program ZIP code area	−.08	0.036	0.050	0.938	1.037	1.146

*Block three*						
Age	.18**					
30–43 versus 18–29		−0.021	0.013	0.954	0.979	1.004
43–81 versus 18–29		−0.107**	0.016	0.871	0.899	0.927
Female	<.01	−0.019	0.012	0.958	0.981	1.005
Race/ethnicity	.30**					
Hispanic versus non-Hispanic white		−0.344**	0.022	0.680	0.709	0.740
Black versus non-Hispanic white		−0.454**	0.025	0.605	0.635	0.666
Other versus non-Hispanic white		−0.109**	0.036	0.836	0.896	0.961
Employed	.13**	0.005	0.012	0.972	0.995	1.019

*Block four*						
Pain a reason for treatment	.01	−0.011	0.012	0.965	0.989	1.013
First methadone treatment	.14**	0.004	0.012	0.980	1.004	1.028
Strong urge to use	.03**	<0.001	0.018	0.966	1.000	1.035
Severe withdrawal	.02**	0.003	0.014	0.976	1.003	1.030
Prescription opioid use in past 30 days	.26**	0.081**	0.016	1.051	1.085	1.119
Heroin use in past 30 days	−.33**	−0.031	0.017	0.939	0.970	1.002

Interval estimates for each predictor have been exponentiated and can be interpreted as how travel distance is multiplied given a one-unit change in the predictor (or a contrast between one level of a categorical predictor and the reference category for that predictor). For predictors with multiple categories (i.e., USA Region, Urbanicity, Age, and Race/Ethnicity), the zero-order correlation is the multiple correlation when log distance is regressed on dummy variables.

*P < .05; **P < .01.

**Table 3:** Multilevel logistic regression model predicting patient travel across state to OTP.

Predictor	95% Confidence interval of the unadjusted odds ratio			95% Confidence interval of the adjusted odds ratio		
	Lower	Odds ratio	Upper	Lower	Odds ratio	Upper
*Block one*						
U.S. Region						
Southeast versus Northeast	0.314	2.516	20.140	0.126	1.051	8.737
Midwest versus Northeast	1.044	11.826	133.970	0.821	8.473	87.475
West versus Northeast	0.003	0.063	1.480	0.002	0.038	0.839
Urbanicity						
250 K − 1M versus Metro > 1 M	1.649	10.959	72.848	1.878	12.081	77.697
<250 K versus Metro >1 M	0.105	1.384	18.160	0.230	3.148	42.997

*Block two*						
Patient ZIP code area	0.921	0.983	1.049	0.918	0.980	1.046
Program ZIP code area	1.024	2.007	3.933	1.317	2.928	6.510

*Block three*						
Age						
30–43 versus 18–29	0.943	1.115	1.320	0.950	1.125	1.332
43–81 versus 18–29	0.891	1.126	1.423	0.900	1.142	1.448
Female	0.872	1.020	1.192	0.837	0.987	1.164
Race/Ethnicity						
Hispanic versus white	0.262	0.559	1.192	0.266	0.570	1.220
Black versus white	0.198	0.416	0.875	0.191	0.402	0.847
Other versus white	0.196	0.403	0.830	0.195	0.403	0.829
Employed	0.778	0.911	1.068	0.767	0.906	1.072

*Block four*						
Pain a reason for treatment	0.882	1.032	1.209	0.880	1.032	1.211
First methadone treatment	0.722	0.849	0.997	0.734	0.868	1.026
Strong urge to use	0.667	0.861	1.112	0.676	0.895	1.185
Severe withdrawal	0.778	0.934	1.123	0.801	0.978	1.194
Prescription opioid use in past 30 days	0.654	0.988	1.493	0.602	0.935	1.450
Heroin use in past 30 days	0.854	1.064	1.327	0.858	1.084	1.369

Patient ZIP code area and program ZIP code area are continuous measures; all other covariates are coded 0, 1.
